# Mouthguard use, hygiene, and maintenance practices among combat and team sports athletes: A comparative study

**DOI:** 10.1371/journal.pone.0317952

**Published:** 2025-01-29

**Authors:** Didem Özkal Eminoğlu, Kamber Kaşali, Cebrail Gençoğlu, Süleyman Ulupinar, Serhat Özbay, Buket Şeran, Ayşegül Türksoy, Fatih Kiyici

**Affiliations:** 1 Department of Periodontology, Faculty of Dentistry, Atatürk University, Erzurum, Türkiye; 2 Department of Biostatistics, Faculty of Medicine, Atatürk University, Erzurum, Türkiye; 3 Department of Coaching Education, Faculty of Sport Sciences, Erzurum Technical University, Erzurum, Türkiye; 4 Department of Physical Education and Sports, Faculty of Sport Sciences, Erzurum Technical University, Erzurum, Türkiye; 5 Department of Coaching Education, Faculty of Sport Sciences, Ataturk University, Erzurum, Türkiye; Ordu University, TÜRKIYE

## Abstract

This study aimed to investigate the attitudes of combat and team sports athletes towards the use, storage, and hygiene conditions of mouthguards, with the goal of understanding disparities in usage and maintenance practices and their implications for oral health. A cross-sectional study was conducted involving 394 athletes (316 combat athletes and 78 team athletes). Participants completed a 28-question survey assessing their knowledge, attitudes, and practices regarding mouthguard use, hygiene, and maintenance. The survey included questions on demographic data, mouthguard usage habits, cleaning methods, and storage practices. Combat sports athletes exhibited significantly higher rates of mouthguard use (100%) compared to team sports athletes (29.5%) (chi square [χ^2^] = 258.971, p<0.001). Significant differences were also found in cleaning (χ^2^ = 252.195, p<0.001) and storage practices (χ^2^ = 14.195, p = 0.002), with combat athletes demonstrating better adherence to recommended guidelines. Common complaints about mouthguards included difficulties with speaking (χ^2^ = 7.792, p = 0.005) and breathing (χ^2^ = 11.431, p = 0.001), with combat athletes reporting fewer issues. The study highlights the need for increased awareness and education on mouthguard use among team sports athletes. Dentists and coaches play a crucial role in promoting proper mouthguard use, hygiene, and maintenance to prevent sports-related dental injuries. Emphasis should be placed on the benefits of custom-made mouthguards for improved comfort and protection.

## Introduction

The utilization of mouthguards in sports, particularly in those characterized by high levels of physical contact such as team sports and individual combat disciplines, is a well-established method for preventing dental and orofacial injuries [1, 2]. The protective value of mouthguards is supported by a significant body of research, which underscores their effectiveness in absorbing and dissipating the forces of impact, thereby reducing the risk of trauma in sports such as boxing, martial arts, rugby, and hockey [1, 3, 4], since certain sportive activities inherently carry a higher risk of injuries [5] that can be mitigated through the use of these protective devices. Athletes often avoid wearing mouthguards due to issues like poor retention, discomfort, and interference with breathing and speaking [4, 6]. These barriers have been noted alongside perceptions that mouthguards are unnecessary, especially in non-contact sports. Although there are concerns among athletes regarding mouthguard use, such as potential breathing difficulties and unpleasant tastes, a research study by Gebauer et al. [6] demonstrates that custom-made mouthguards do not significantly affect respiratory functions. This evidence suggests that, contrary to common perceptions, these protective devices can be comfortably used by athletes without compromising their performance or breathing, thus enhancing safety in sports without hindering physical capabilities [6].

The actual use, maintenance, and hygiene practices associated with mouthguards among athletes have been identified as areas needing improvement. Compliance with recommended care procedures varies widely, potentially undermining the protective function of these devices [7–10]. This issue is particularly concerning given that poor maintenance can render mouthguards a nidus for pathogens, thereby posing a risk to both oral and general health [11].

The disparity in mouthguard use between team and individual sports athletes highlights an area ripe for investigation. Factors such as educational outreach, cultural attitudes, and sport-specific dynamics may influence these discrepancies [12]. Moreover, the impact of educational interventions on improving mouthguard hygiene habits represents a significant area for research, given the varying levels of awareness across different sports and competitive levels.

Concerns about the microbiological safety of mouthguards have been growing, with studies identifying a diverse array of microorganisms that can colonize mouthguards if they are not properly maintained. This contamination can lead to various health issues, from minor oral infections to more significant systemic conditions [10, 11, 13]. Despite this knowledge, adherence to recommended cleaning and storage practices remains suboptimal among many athletes, underscoring a critical gap in sports health education [12].

Given these considerations this research intends to uncover the underlying factors influencing these practices and increasing the awareness on improving mouthguard hygiene. Specifically, we hypothesize that there exists a significant difference in mouthguard usage and maintenance practices between athletes participating in team sports and those engaged in individual sports.

By addressing this hypothese, our study aims to contribute valuable insights to the existing body of knowledge, advocating for the development of more effective strategies to promote the proper use and maintenance of mouthguards, ultimately enhancing the oral health and safety of athletes across various sports disciplines.

## Materials and methods

### Study design and ethical approvals

This cross-sectional study was conducted on individuals who were actively engaged in sports at a professional level. The survey was performed in Turkey between 25.02.2024 and 25.04.2024. The ethics committee approval of the study was received from Atatürk University Ethic Committee (Decision Date: 22.02.2024; Session Number: 02/2024; Decision No: 12). All participants and the parents of the child athletes were given detailed information about the goals and design of the current study and signed informed consent forms.

### Sample size and study population

With reference to the article by Dursun et al. [13]; the sample size calculation was made in terms of the knowledge of team and combat sports players about the mouthguard, it was determined with the G*Power program that there should be at least 73 participants in each group at 80% power and 95% confidence level in order for a difference of 22% to be significant if the knowledge of team sports is 21%, while this rate is 43% in combat sports.

In our study, 394 elite level athletes, of which 316 (80.2%) were combat athletes (boxing, kickboxing, karate, Muay Thi) and 78 (19.8%) were team athletes (volleyball, basketball, rugby, football, and ice hockey) participated. All of the athletes who participated in the questionnaire were from Turkey consisted of players who participated professional competitions in 2024 and has been practicing at least 3 years of sports dicipline. A targeted questionnaire concerning understanding, experiences, and behaviors related to mouthguard usage was administered to sports participants aged 16 to 50 years. Participation to the survey was voluntary, and there were no consequences or benefits associated with it.

Participants lacking a consistent professional sports background, those with systemic diseases that avoided the use of mouthguards, and individuals with inadequate cognitive abilities to read, comprehend, and respond to the questions were excluded from the study.

### Development and execution of survey questionnaires

By evaluating the questionnaires from similar studies in the literature [13–20], a new specific questionnaire was created including the knowledge levels of athletes about mouth guards and their knowledge about the use of mouth guards. The questionnaire consists of 7 “descriptive”, 8 “attitudes of mouthgard usage” 5 “mouthguard maintenance and hygiene”, and 8 “complaints of mouthgard using” questions, totaling 28 questions. The questionnaire was distributed to active professional athletes in Turkey via the online survey platform "docs.google" [21]. Participant confidentiality was maintained with no names recorded on the questionnaire.

### Statistical analysis

Statistical analyses were conducted using IBM SPSS Statistics version 20 (Armonk, NY, USA). Data were presented as mean, standard deviation, median, minimum, maximum, percentage, and number. The normality of continuous variables was assessed using the Shapiro-Wilk test, Kolmogorov-Smirnov test, Q-Q plot, skewness, and kurtosis. For comparisons between two independent groups, the Independent Samples t-test was used when the normal distribution assumption was met; otherwise, the Mann-Whitney U test was employed. The one-sample chi-square test was utilized for comparing two proportions. For 2x2 comparisons of categorical variables, the Pearson chi-square test was applied if the expected value was (>5), the chi-square with Yates’ correction was used if the expected value was between 3 and 5, and Fisher’s Exact test was performed if the expected value was (<3). For comparisons involving categorical variables larger than 2x2, the Pearson chi-square test was used when the expected value was (>5), and the Fisher-Freeman-Halton test was employed when the expected value was (<5). The level of statistical significance was set at p<0.05.

## Results

A total of 394 athletes participated in our study, comprising 316 (80.2%) combat sports athletes and 78 (19.8%) team sports athletes. Among the participants, 285 (72.3%) were male and 109 (27.7%) were female. The overall mean age of the athletes was 21.66 ± 7.41 years; specifically, 21.86 ± 8.02 years for combat sports athletes and 20.86 ± 4.07 years for team sports athletes (p = 0.272, Z = -1.099). A statistically significant difference was observed in the education levels between combat and team sports athletes (p<0.001, Chi-Square [χ^2^] = 24.856) (Table 1).

**Table 1:** Demographic data of the athletes.

		Combat Sports		Team Sports		
		Mean ± sd	Median (25–75 Percentile)	Mean ± sd	Median (25–75 Percentile)	p
**Age**		21,86 ± 8,02	19,0 (17,0–23,0)	20,86 ± 4,07	20,5 (17,0–23,0)	0,272 ^Z^
** **		**N**	**%**	**N**	**%**	**p**
**Sex**	Male	222	0,70	63	0,81	0,063 ^***χ^2^***^
	Female	94	0,30	15	0,19	
**Education Level**	High School	216	0,68	32	0,41	**<0,001** ^χ^2^^*
	University	82	0,26	44	0,56	
	Master-PhD	18	0,06	2	0,03	

Z: Mann-Whitney U Test; χ^2^ = Pearson Chi-Square Test; sd: Standart deviation; * = Statistical significant p<0,05.

The combat sports athletes who participated in the study consisted of 41 (13%) boxers, 137 (43.4%) kickboxers, 39 (12.3%) karate athletes, 36 (11.4%) Muay Thai athletes, and 63 (19.9%) taekwondo athletes. The team sports athletes included participants from volleyball, basketball, rugby, football, and ice hockey disciplines.

Table 2 demonstrated that there were statistically significant differences between combat sports athletes and team sports athletes in terms of being an international athlete (p = 0.004, χ^2^ = 8.267), having medals in international competitions (p = 0.015, χ^2^ = 8.474), and training age (p = 0.020, Z = -2.331). However, no significant differences were observed in training hours per week (p = 0.561, Z = -0.582).

**Table 2:** Demographic data of the athletes.

		Combat Sports		Team Sports		
		Mean ± sd	Median (25–75 Percentile)	Mean ± sd	Median (25–75 Percentile)	p
**Training age**		7,3 ± 6,5	5,0 (2,0–10,0)	7,9 ± 4,5	8,0 (4,0–11,0)	**0,020** ^**Z**^ *
**Training hours in a week**		8,4 ± 5,4	6,0 (5,0–11,0)	7,9 ± 5,2	6,0 (4,0–10,0)	0,561^Z^
** **		**N**	**%**	**N**	**%**	**p**
**International athlete status**	No	231	0,73	44	0,56	**0,004** ^χ^2^^*
	Yes	85	0,27	34	0,44	
**Do you have any medals in international competitions?**	No	17	0,05	8	0,1	**0,015** ^χ^2^^*
	Yes	68	0,22	26	0,33	
	Non-international	231	0,73	44	0,56	

Z: Mann-Whitney U Test; chi-square: χ^2^ = Pearson Chi-Square Test; sd: Standart deviation: * = Statistical significant p<0,05.

Table 3 displays the responses of combat and team sports athletes to the survey questions regarding mouthguard usage. There were statistically significant differences between combat and team sports athletes in their responses to questions assessing their knowledge about mouthguards (Q1-Q3) (p<0.001, χ^2^ = N/A for Q1, χ^2^ = 216.201 for Q2, χ^2^ = 196.683 for Q3). Significant differences were also found between combat and team sports athletes in their responses to questions Q4 (p<0.001, χ^2^ = 258.971), Q6 (p<0.001, χ^2^ = 41.698), and Q8 (p<0.001, χ^2^ = 39.611) regarding mouthguard usage. However, no significant differences were observed in the responses to questions Q5 (p = 0.973, χ^2^ = 0.001) and Q7 (p = 0.509, χ^2^ = 1.303).

**Table 3:** Responses of combat and team sports athletes to mouthguard usage survey questions.

		Combat Sports		Team Sports		
		N	%	N	%	p
**Q1. Do you know what a mouthguard is?**	No	0	0,0%	5	6,4%	**<0,001** ^**δ**^ *
	Yes	316	100,0%	73	93,6%	
**Q2. Have you ever been informed about the use of mouthguards?**	No	0	0,0%	47	60,3%	**<0,001** ^χ^2^^*
	Yes	316	100,0%	31	39,7%	
**Q3. If informed, where did you receive this information?**	I was not informed	0_a_	0,0%	47_b_	60,3%	**<0,001** ^**ϕ**^ *
	Coach/Sports Trainer	302_a_	95,6%	22_b_	28,2%	
	Dentist	7_a_	2,2%	4_a_	5,1%	
	Social Media/Web/TV, etc.	7_a_	2,2%	5_a_	6,4%	
**Q4. Do you use a mouthguard?**	No	0	0,0%	55	70,5%	**<0,001** ^χ^2^^*
	Yes	316	100,0%	23	29,5%	
**Q5. What type of mouthguard do you use?**	Stock/‘boil and bite’	205	64,9%	15	65,2%	0,973 ^**χ^2^**^
	Costum	111	35,1%	8	34,8%	
**Q6. Do you like wearing a mouthguard?**	No, I don’t like	156	49,4%	70	89,7%	**<0,001** ^χ^2^^*
	Yes, I like	160	50,6%	8	10,3%	
**Q7. When do you use your mouthguard?**	Only in competitions	154	48,7%	11	47,8%	0,509^**ϕ**^
	Only in trainings	6	1,9%	1	4,3%	
	Both in competitions and trainings	156	49,4%	11	47,8%	
**Q8. Would you refuse to participate in a sports competition or training session without your mouthguard?**	No	155	49,1%	69	88,5%	**<0,001** ^χ^2^^*
	Yes	161	50,9%	9	11,5%	

χ^2^ = Pearson Chi-Square Test; δ = Fisher’s Exact test; ^**ϕ**^ = Fisher-Freeman-Halton testi; ^a,b,c^: post-hoc test results; Q: Question; * = Statistical significant p<0,05.

When examining the responses to questions about mouthguard cleaning habits as described in Table 4 (Q9, Q10, Q11), statistically significant differences were found between combat athletes and team athletes. For Q9, which asked if they clean their mouthguards, 98.4% of combat athletes reported cleaning their mouthguards compared to 82.6% of team athletes (p<0.001, χ^2^ = 252.195). Regarding Q10, which inquired about the method used to clean their mouthguards, the majority of combat athletes (63.6%) cleaned their mouthguards under running water only, while 52.2% of team athletes did the same. Additionally, 28.5% of combat athletes used toothpaste and a toothbrush compared to 21.7% of team athletes. A smaller percentage used special cleaning agents (6.3% combat athletes and 8.7% team athletes). Notably, 17.4% of team athletes reported not cleaning their mouthguards at all, whereas this was reported by only 1.6% of combat athletes (p = 0.004, χ^2^ = 12.274). For Q11, which addressed the frequency of cleaning, significant differences were found (p = 0.035, χ^2^ = 12.018). Among combat athletes, 23.1% cleaned their mouthguards before each use, 20.3% after each use, and 38.0% both before and after each use. In contrast, 26.1% of team athletes cleaned their mouthguards before each use, 17.4% after each use, and 34.8% both before and after each use. Weekly cleaning was reported by 4.7% of combat athletes, while none of the team athletes reported this frequency. Cleaning once a month was reported by 2.8% of combat athletes, whereas none of the team athletes reported this frequency. Cleaning from competition to competition was reported by 9.5% of combat athletes and 4.3% of team athletes. Lastly, 17.4% of team athletes did not clean their mouthguards at all, compared to only 1.6% of combat athletes. There was also a statistically significant difference in the methods of storing mouthguards (Q12) between the two groups (p = 0.002, χ^2^ = 14.195). Both groups showed a higher preference for storing their mouthguards in a box/container. Specifically, 91.5% of combat athletes preferred storing their mouthguards in a box/container, while team athletes were more likely to store their mouthguards in a special solution (17.4%) or a plastic bag (13.0%). Additionally, there was a statistically significant difference in the frequency of mouthguard replacement (Q13) between combat athletes and team athletes (p<0.001, χ^2^ = 19.311). The significant differences were observed in the responses “I change it every month” and “I do not change it unless it is lost or damaged.” Among combat athletes, 1.3% reported changing their mouthguards every month, compared to 13% of team athletes. Conversely, 53.5% of combat athletes indicated that they do not change their mouthguards unless they are lost or damaged, whereas this figure was 17.4% among team athletes.

**Table 4:** Responses of combat and team sports athletes to mouthguard maintenance and hygiene.

		Combat Sports		Team Sports		
		N	%	N	%	p
**Q9. Do you clean your mouthguard?**	No	5	1,6%	4	17,4%	**<0,001** ^χ^2^^*
	Yes	311	98,4%	19	82,6%	
**Q10. What method do you use to clean your mouthguard?**	Under running water only	201_a_	63,6%	12_a_	52,2%	**0,004** ^ϕ^ *
	With special cleaning agents	20_a_	6,3%	2_a_	8,7%	
	With toothpaste and a toothbrush	90_a_	28,5%	5_a_	21,7%	
	I do not clean it	5_a_	1,6%	4_b_	17,4%	
**Q11. How often do you clean your mouthguard?**	Before each use	73_a_	23,1%	6_a_	26,1%	**0,035** ^ϕ^ *
	After each use	64_a_	20,3%	4_a_	17,4%	
	Before and after each use	120_a_	38,0%	8_a_	34,8%	
	Once a week	15_a_	4,7%	0_a_	0,0%	
	Once a month	9_a_	2,8%	0_a_	0,0%	
	From competition to competition	30_a_	9,5%	1_a_	4,3%	
	I do not clean it	5_a_	1,6%	4_b_	17,4%	
**Q12. How do you store your mouthguard?**	I store it in a box/container.	289_a_	91,5%	15_b_	65,2%	**0,002** ^ϕ^ *
	I store it in a special solution.	14_a_	4,4%	4_b_	17,4%	
	I store it in a plastic bag.	8_a_	2,5%	3_b_	13,0%	
	I store it without putting it in a box/container.	5_a_	1,6%	1_a_	4,3%	
**Q13. How often do you replace your mouthguard?**	Every month	4_a_	1,3%	3_b_	13,0%	**<0,001** ^ϕ^ *
	Every 3 months	36_a_	11,4%	5_a_	21,7%	
	Every 6 months	48_a_	15,2%	6_a_	26,1%	
	Once a year	47_a_	14,9%	4_a_	17,4%	
	Every 2 years or more	12_a_	3,8%	1_a_	4,3%	
	I do not change it unless it is lost or damaged.	169_a_	53,5%	4_b_	17,4%	

χ^2^ = Pearson Chi-Square Test; δ = Fisher’s Exact test; ^ϕ^ = Fisher-Freeman-Halton testi; ^a,b,c^: post-hoc test results; Q: Question; * = Statistical significant p<0,05.

Table 5 displays the complaints of athletes during mouthguard use, highlighting significant differences between combat sports and team sports athletes. A significant difference was found in the response to Q14, where 22% of combat athletes reported no complaints compared to 15% of team athletes (p<0.001, χ^2^ = 29.545). For Q15, 20% of combat athletes reported difficulty speaking, whereas this was significantly higher at 37% for team athletes (p = 0.005, χ^2^ = 7.792). Similarly, Q16 showed that 16% of combat athletes experienced difficulty breathing, compared to 20% of team athletes, indicating a significant difference (p = 0.001, χ^2^ = 11.431). Regarding Q17, 5% of combat athletes reported that mouthguard use reduced their performance during training, while none of the team athletes reported this issue (p = 0.005, χ^2^ = 7.727). For Q18, 8% of combat athletes and 7% of team athletes reported experiencing dry mouth, showing a significant difference (p = 0.014, χ^2^ = 6.048). Additionally, Q19 revealed that 10% of combat athletes were disturbed by the taste and smell of the mouthguard compared to 1% of team athletes, indicating a significant difference (p<0.001, χ^2^ = 14.321). Lastly, for Q20, 16% of combat athletes reported nausea and the feeling of vomiting, whereas 12% of team athletes reported the same (p<0.001, χ^2^ = 16.654). There was no significant difference for Q21 regarding the perception of looking bad due to wearing a mouthguard (p = 0.508, χ^2^ = 0.438).

**Table 5:** Complaints of athletes during mouthguard use.

	Combat Sports		Team Sports		
	N	%	N	%	p
**Q14. I do not have any complaints**	127	0,22	6	0,15	**<0,001** ^χ^2^^*
**Q15. I have difficulty speaking**	113	0,20	15	0,37	**0,005** ^χ^2^^*
**Q16. I have difficulty breathing**	91	0,16	8	0,20	**0,001** ^χ^2^^*
**Q17. It reduces my performance during training**	29	0,05	0	0,00	**0,005** ^**δ**^ *
**Q18. It causes dry mouth**	44	0,08	3	0,07	**0,014** ^**δ**^ *
**Q19. I am disturbed by its taste and smell**	58	0,10	1	0,02	**<0,001** ^**δ**^ *
**Q20. It causes nausea and the feeling of vomiting**	90	0,16	5	0,12	**<0,001** ^χ^2^^*
**Q21. It makes me look bad**	21	0,04	3	0,07	0,508^δ^

χ^2^ = Chi-Square Test; δ = Fisher’s Exact test; ^a,b,c^: post-hoc test results; Q: Question; * = Sta

## Discussion

This study investigated the attitudes of combat and team sports athletes towards the use, storage, and hygiene conditions of mouthguards. One of the key findings was the significant difference in mouthguard usage between the two groups, with all combat athletes using mouthguards, while only 29.5% of team sports athletes reported using them. This aligns with previous research, which showed low usage rates among team sports athletes despite awareness of the protective benefits [13, 20].

Traumatic oral injuries are a distinct possibility during recreational and sporting activities [17]. A considerable number of dental and facial injuries have been incurred as a consequence of widespread participation and intense competition in organized sports [22]. The physical style of sport, fast speed, and close contact enhance the chance of trauma [18]. Dental injuries associated with sports frequently arise from incidents involving hand or forearm contact with the face, collisions with fellow athletes, or falls [23]. Prior investigations involving diverse cohorts of amateur and professional athletes from various nations have established that the incidence of sports-related dental trauma varies between 8% and 45% [24–26]. Soft-tissue lacerations, tooth fractures, dislocations, avulsions, hematomas, jaw dislocations, jaw fractures, jaw discomfort, and severe craniocerebral traumas are all considered oral injuries [19]. Frequently, injuries to the orofacial region result in severe complications accompanied by substantial post-treatment expenses. Tooth loss, tooth color alteration, pulp necrosis, and infection were the most prevalent dental complications [14].

Use of appropriate protective equipment, including facemasks, mouthguards, and helmets, may aid in the reduction of the frequency and severity of sports-related injuries. The athletic mouthguard, also known as a mouth protector, is often regarded as the key device for reducing dental and facial injuries. Mouthguards typically are designed to cover surfaces of the maxillar teeth and gingivae to minimize the transmitted forces to the hard or soft tissues. Mouthguards in contact sports can decrease the occurrence of dental injuries by as much as 90% [13]. In sports where athletes consistently wore mouthguards, the risk of dental injuries was significantly lower [14]. However, up to 25% of dental injuries might happen despite the use of a mouthguard; such injuries are relatively minor (e.g., socket hemorrhage and tooth luxation). This study showed that a high proportion of participants reported knowing what mouthguards are in both combat and team sports. Despite this knowledge, usage rates were significantly higher among combat athletes compared to team sports athletes. This disparity can be attributed to the mandatory requirement of mouthguards in combat sports versus the optional use in many team sports. Nonetheless, the wearing of mouthguards, while advised, is not mandated in numerous sports like basketball and soccer [13]. Lehl also [27] stated that athletes’ use of protective mouthguards varies greatly in different sports diciplines. Kujala et al. [28] reported that injury rates were higher in sports characterized by more frequent and intense physical contact. This could provide an alternative reason for the difference in athletes’ preferences for mouthguard usage across different sports disciplines. Cornwell et al. [16] revealed a significant cognitive conflict between the opinions of players and their reported behaviors. Although nearly all athletes (90%) recognized the importance of mouthguards in preventing injuries, the actual usage of mouthguards was low (25%), except among those with a prior orofacial injury. Previous experience of orofacial injuries may be regarded as a contributing factor to the variation in mouthguard use. The insufficient precautions in team sports like baseball, basketball, soccer, softball, and volleyball to safeguard athletes from injuries underscore the need for better enforcement of protective equipment use [29]. The extent of knowledge about and use of mouthguards among athletes was determined with athletes from various countries such as Turkey [30], Poland [31], Germany and United States [32], Croatia [14], Australia [18]. These studies conducted in different countries delivered both similar and contrasting conclusions. A contributing factor to this circumstance may be culture-specific attitudes. Hoffmann et al. [32] stated that mouthguards are significantly less widely accepted in Germany than to the United States.

Bergman et al. [14] identified the lack of recommendations as a primary reason for not wearing mouthguards, accounting for 29% of cases. In our study, 60.3% of team athletes were not informed about mouthguard use, correlating with their low usage rate of 29.5%. Raising awareness among athletes, coaches, and the dental community is crucial [23]. Notably, 95.6% of combat athletes and 28.2% of team athletes received recommendations from coaches or sports trainers. Ilia et al. [18] highlighted family and friends as primary sources of guidance.

A prior study showed that 76.9% of athletes who consistently wore mouthguards did so based on their dentist’s recommendation, highlighting the dentist’s influence on behavior. In contrast, only a small percentage of participants in our study (combat athletes, 2.2%; team athletes, 5.1%) received recommendations from their dentists [4]. Similarly, Ma [19] reported that dentists ranked behind athletes, media, teammates, and coaches in influencing mouthguard use. Therefore, it is essential for dentists and other healthcare professionals to educate players, parents, and coaches to promote mouthguard use in both professional and amateur sports, with a particular emphasis on adolescents.

Studies have identified communication difficulties (27.9%), breathing issues (21.2%), and aesthetic concerns (19.1%) as primary reasons for not using mouthguards [33]. Bergman et al. [14] reported that 24% of players found mouthguards uncomfortable with similar complaints of poor fit and difficulty breathing noted by Newsome et al. [4]. Elite female athletes and basketball players also reported speech and breathing problems [19]. In our study, common complaints included difficulty speaking (20%-37%) and breathing (16%-20%), with most combat athletes (22%) experiencing no issues, likely due to the mandatory use of mouthguards in their sports. Despite reported discomfort, studies have shown that custom-made mouthguards do not negatively impact performance, unlike boil-and-bite types, which can be uncomfortable and impede breathing [34]. Gawlak et al. [35] also indicated that mouthguards do not significantly affect the basic functions of the stomatognathic system. To address comfort issues, customized mouthguards are recommended as they offer a closer fit to the dental arch, reducing breathing and speaking challenges while providing superior protection compared to standard mouthguards [34]. Despite their benefits, our study found that most athletes (combat sports, 64.9%; team sports, 65.2%) used "boil and bite" mouthguards, likely due to their lower cost and lack of awareness about the advantages of custom-made options. Athletes wearing mouthguards are not usually adequately informed by dentists of the extensive variety of mouthguards, which vary in construction, energy absorption, injury prevention capabilities [36]. Guevara and Ranalli [37] stated that boil-and-bite mouthguards are the most frequently used because to cost-effectiveness and ease of adaptation. The increased availability of custom-made mouthguards through dental organizations underscores the importance of dentists advocating for their use. Custom-made mouthguards provide a better fit, greater comfort, and improved protection, making it crucial for dentists to encourage athletes to choose these over standard options. An increasing number of general dentists are now offering custom-fabricated mouthguards to their patients. Nonetheless, a considerable proportion of dentists do not offer this service to their regular consumers. Addressing complaints regarding cost for custom-made versus store-bought is essential for dentists and athletes [38]. The high cost of custom-made mouthguards has emerged as a barrier limiting their usage [39]. Patrick et al. [40] remarked that while athletes are willing to invest substantial amounts in the latest equipment, minimal consideration is afforded to safeguarding the teeth, mouth, and skull from recurrent concussions. It is anticipated that the use of stock mouthguards will decline in favor of custom-made mouthguards, consequently decreasing the occurrence of oral trauma in sports. Reducing the cost implications through potential solutions (e.g. subsidies, insurance coverage) could lead to a preference for the use of custom-made mouthguards.

Studies recommend that mouthguards should be worn during both practices and competitions [1]. The lower usage rate during training displays that players perceive less danger compared to competitive situations. However, much of the time in contact sports is dedicated to full-contact practice, where acciddents and injuries can also occur. This indicates a lack of understanding among players about the risks during training and the importance of wearing mouthguards consistently [18]. Training can be intense, especially when competing for team positions, and players often spend more time training than competing, increasing their injury risk [17]. A previous study found that 60% of individuals who experienced orofacial injuries without a mouthguard later adopted their use, with 40% wearing them during both training and competitions [14]. Therefore, it is essential for dentists, sports physicians, and coaches to promote the use of mouthguards in all settings [12].

Earlier studies have shown that mouthguards used by athletes contain a diverse array of bacteria, including opportunistic and pathogenic strains, as well as fungi such as yeasts and molds [41]. These microorganisms, linked to various infections like oral thrush, meningitis, urinary tract infections, peritonitis, periodontal disease, wound infections, and exercise-induced asthma, have been found in sports mouthguards [42]. Scanning electron microscopy (SEM) revealed that mouthguards have a similar porosity to dentures, allowing pathogens to reside on surfaces and within pores, forming intricate biofilms [43]. Additionally, used mouthguards can develop sharp edges causing oral lacerations, providing an entry point for germs into the bloodstream and respiratory system [44]. Proper storage and hygiene are crucial to minimize contamination risks. This study focuses on optimal cleaning and storage conditions for athletes’ mouthguards, with previous research suggesting weekly replacement [44]. In our study, 98.4% of combat sports athletes and 82.6% of team sports athletes reported cleaning their mouthguards, with most doing so before and after each use (combat sports, 38.0%; team sports, 34.8%). Despite these cleaning practices, the frequency of mouthguard replacement varied significantly. Only 1.3% of combat athletes and 13.0% of team athletes replaced their mouthguards every month, while 53.5% of combat athletes and 17.4% of team athletes did not change their mouthguards unless they were lost or damaged. This indicates a need for increased awareness about the importance of regular replacement to ensure optimal hygiene and protection.

It is important to disinfect sports mouthguards to prevent changes in the oral microbiota [45]. Daily sanitization of mouthguards is classified as category A in the Strength of Recommendation Taxonomy (SORT) evidence assessment system [46, 47]. There is insufficient disclosure in the media and professional sports of the importance for sanitation and the cleansing of mouthguards. Mouthguards can serve as a possible source of contamination and disease transmission for professional athletes [48]. Mouthguards, like dentures, have a high degree of porosity, creating an ideal habitat for microorganisms. These bacteria infiltrate the pores over time and thrive on moisture and nourishment from the athlete. Cleaning treatments often struggle to effectively penetrate these microscopic pores [44]. Several substances, including sterile distilled water, hydrogen peroxide, physiological saline solution, fluoride toothpaste, 0.5% sodium hypochlorite solution, Oral Care Foam™, and Bite Sept™, have been tested for their antimicrobial effects, but no gold standard method for decontamination exists [45]. Disinfectants can reduce microorganisms but may weaken the mouthguard’s structure over time. Ogawa et al. [49] found that using sterilized water and a well-ventilated setting is effective for sanitary storage. In our study, the majority of combat athletes (63.6%) and team sports athletes (52.2%) rinsed their mouthguards only with water, while 28.5% of combat athletes and 21.7% of team sports athletes used toothpaste and a toothbrush. However, these methods do not eliminate attached microbes [49] and brushing can damage the mouthguard’s surface [50]. Although there is no standard cleaning method [51], studies have shown that chemical cleaning methods are more effective for sanitization of the mouthgards because mouthguards deteriorate over time and have morphologically challenging areas to clean [49, 51]. Trenton et al. [52] stated in the "National Athletic Trainers’ Association Position Statement" that athletes are encouraged to disinfect the mouthguard with a mild antibacterial solution and rinse it well in lukewarm water both before to and following use. Spraying antiseptic solutions during decontamination has many benefits, including convenience of use, economy of dose, application of a new dose at each decontamination, and ease of transporting to practice or competition [53]. The required concentration of the spray must be applied for a specific duration to achieve optimal disinfection, which varies based on the disinfectant and the microorganisms targeted [54]. Tanabe et al. [51] found that the disinfecting spray was effective after a 60-second exposure to the specimen piece. A disinfectant spray including cetylpyridinium chloride (CPC), used in nasal sprays, troches, and mouthwashes, is suitable for chemical cleaning due to its biological safety and antibacterial properties [51]. Silver-containing materials exhibit a wide range of antibacterial properties; silver can interact with bacterial proteins and enzymes, sustaining significant antibacterial efficacy and notable heat resistance [55]. Commercially accessible antibacterial coating agents, such as silver particles, suppress bacterial colonization. Nonetheless, due to their intricate application and limited sustainability, these compounds are not extensively utilized [56]. A study suggested that an agent named TAE may have clinical use as an antibacterial cleaning material for mouthguards [55].

According to previous studies, mouthguards are often used repeatedly for several months or years, leading to an increase in the quantity and variety of bacteria with each use [48, 57]. The SORT evidence rating has classified the replacement of the protective athletic mouthguard as B category when it becomes pointed or jagged, or when there is an oral irritation or ulceration [46, 47]. The athlete must assess the mouthguard daily for proper fit and any damage, such as tears in the material or lack of resilience. The mouthguard must be replaced if it is loose or impaired [52]. Previous research indicated an increase in bacterial contamination regardless of whether athletes used the same mouthguard throughout a football season or replaced it with a new one [42]. Our study found that 53.5% of combat athletes do not change their mouthguards unless they are lost, which could lead to increased bacterial contamination and potential health risks. In contrast, 21.7% of team sports athletes replace their mouthguards every 3 months, and 26.1% replace them every 6 months. Daily storage and cleaning practices vary widely and are often inadequate [48, 51]. The storage conditions of mouthguards are often not hygienic enough. Regarding preservation, numerous athletes carelessly leave their mouthguards without considering the risk of infection [42]. The optimal storage method involves using containers with perforations for ventilation and inserting a napkin to remove liquid residue [45, 58]. In the present study, 91.5% of combat sports athletes and 65.2% of team sports athletes stored their mouthguards in a plastic box. Proper maintenance of mouthguards is crucial and can be achieved by following simple hygiene measures [44]. Trenton et al. [52] suggested in the "National Athletic Trainers’ Association Position Statement" that athletes need to be advised to preserve the mouthguard in a hygienic solid, aired plastic container. They also suggested that mouthguards should not be left to extended exposure to direct sunlight or heat sources, as this may lead to deformation and diminish their protective efficacy. Ensuring athletes adopt these practices is essential for preventing bacterial contamination and maintaining oral health.

## Conclusions

This study highlights the critical importance of mouthguard use, proper maintenance, and regular replacement among athletes. Despite the well-documented benefits, a significant disparity exists between combat athletes, who universally use mouthguards, and team sports athletes, among whom usage is much lower. The influence of dentists and coaches is paramount in encouraging mouthguard use, yet many athletes still lack proper guidance. Custom-made mouthguards offer superior comfort and protection compared to "boil and bite" types, yet the latter remains more commonly used due to cost and awareness issues. Proper storage and hygiene practices are essential to prevent bacterial contamination, but many athletes do not adhere to recommended practices. To reduce sports-related dental injuries, ongoing education and advocacy from dental professionals and coaches are crucial.
